# *In situ* reconstruction of an infected infrarenal aortic pseudoaneurysm and arteriovenous fistula with self-made pericardium graft

**DOI:** 10.1093/icvts/ivad100

**Published:** 2023-06-26

**Authors:** Guangmin Yang, Tao Sun, Hongwei Chen, Leiyang Zhang

**Affiliations:** Department of Thoracic and Cardiovascular Surgery, Nanjing First Hospital, Nanjing Medical University, Nanjing, China; Department of Thoracic and Cardiovascular Surgery, Nanjing First Hospital, Nanjing Medical University, Nanjing, China; Department of Thoracic and Cardiovascular Surgery, Nanjing First Hospital, Nanjing Medical University, Nanjing, China; Department of Thoracic and Cardiovascular Surgery, Nanjing First Hospital, Nanjing Medical University, Nanjing, China

**Keywords:** Abdominal aortic pseudoaneurysm, Artery-vein fistula, Pericardial patch, Antibiotic therapy, *In situ* reconstruction

## Abstract

Infectious aortic disease is a challenging life-threatening disease in cardiovascular surgery. A 70-year-old man patient presented with an infected infrarenal aortic pseudoaneurysm and right iliac artery- left iliac vein fistula (arteriovenous fistula). He underwent total infected tissues excision, debridement, *in situ* reconstructions of the aorta using a self-made pericardium graft with omental coverage and arteriovenous fistula patch repair to prevent leakage. One-year follow-up revealed the absence of clinically relevant infection with patency of the graft and the absence of biochemical inflammatory markers.

## INTRODUCTION

Infectious pseudoaneurysms of the abdominal aorta are rarer than endograft infections. However, complications, including septicopyemia and rupture, are usually dramatic, resulting in high morbidity and mortality [1]. This report describes a case of *in situ* reconstruction for a native infected abdominal aortic pseudoaneurysm with right iliac artery firstly—left iliac vein fistula [arteriovenous fistula (AVF)].

### Surgical technique

**Figure 1:**
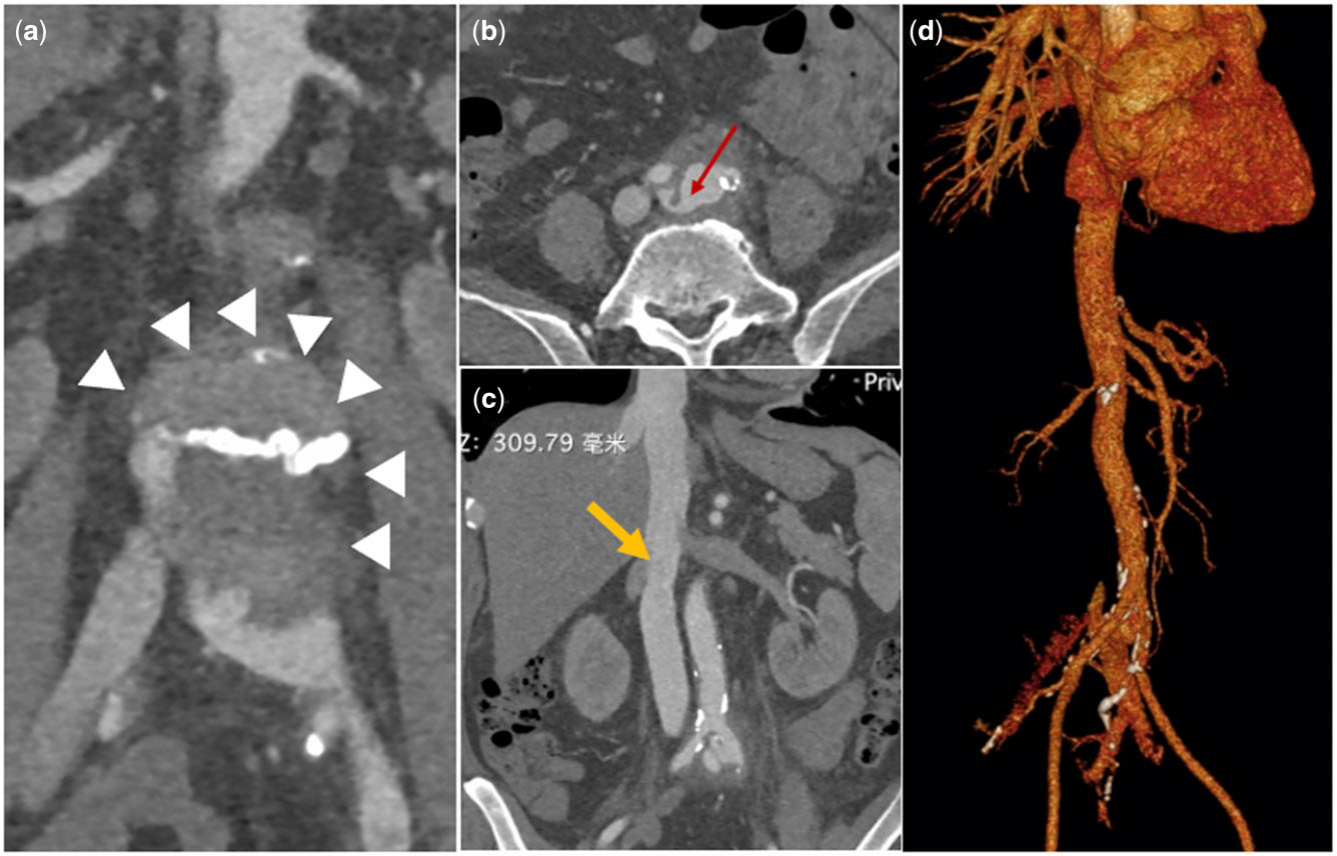
Computed tomography angiography (CTA) shows aortic pseudoaneurysm (**a**, arrowhead) with arterio-venous fistula (**b**, arrow). Inferior vena cava (**c**, arrow) in the coronal view of CTA. The fistula between the right iliac artery and left iliac vein (**d**).

A 70-year-old man was diagnosed with an impending rupture of an infected infrarenal abdominal aortic pseudoaneurysm with AVF (Fig. 1). He presented with a high fever (39.0°C) and left lower extremity swelling for several weeks. Blood cultures demonstrated staphylococcus aureus. He was treated with vancomycin (2 g/day), sulperazone (2–4 g/day) and nutrition support therapy. Three weeks after admission, blood culture results had repeatedly been negative. He consented to undergo open surgical repair.

**Figure 2:**
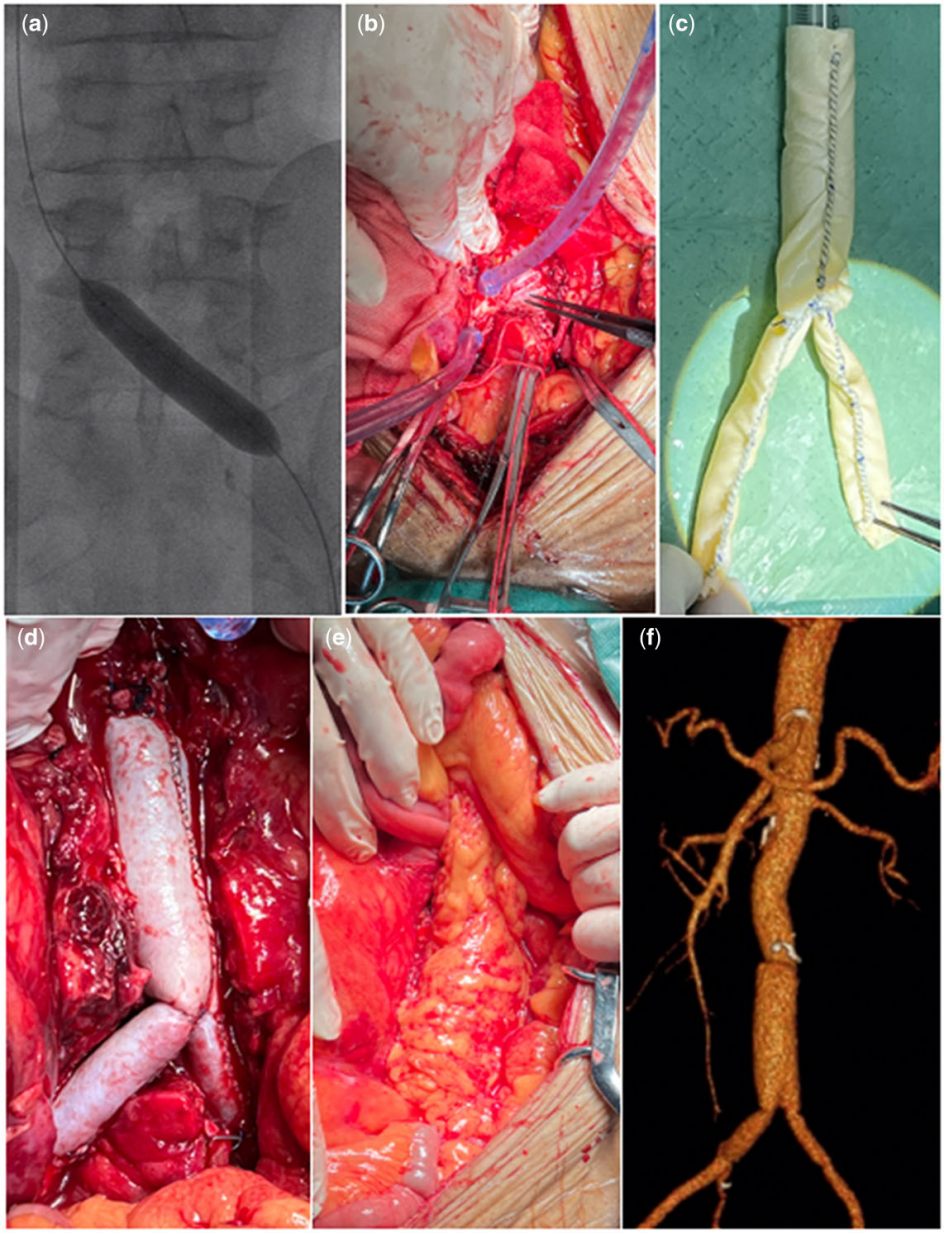
An 8-mm balloon-inflatable tip was used to left iliac vein blocking (**a**). The fistula was repaired by pericardial path (**b**). Bifurcated tube graft made of a bovine pericardial path before (**c**) and after implantation (**d**). The greater omentum was used to cover the vascular graft (**e**). Bifurcate graft on 1-year follow-up imaging (**f**).

Under general anaesthesia, one surgeon performed a laparotomy. Construction of the bifurcated bovine pericardium graft was completed on the back table. The graft size was constructed based on the required anatomy from measurements made from the preoperative CTA image. The pericardial bifurcate graft was prepared with two 8 cm × 10 cm bovine pericardial patch. The main body of the bovine graft was sewn to form a tube using a polypropylene 4–0 running suture. The suture was interrupted every 2 cm. Bifurcated grafts were constructed in a similar manner (Fig. 2c). An 8-mm balloon-inflatable tip was used for a short period of left iliac vein blocking (Fig. 2a) to prevent AVF bleeding during the operation. After all, the procedure was performed well; systemic heparin was given intravenously before placing the clamps. After cross-clamping the infrarenal abdominal aorta and iliac arteries, the aneurysm sac was opened. The reconstruction of the AVF was not feasible due to the necrotic tissues and the infected environment. We performed a fistula repair with a 3 cm × 4 cm pericardial patch (Fig. 2b). All infected tissues and necrotic aortic walls were removed. An *in situ* reconstruction with a self-made pericardial graft was performed (Fig. 2d); the excellent omentum wrap was used to cover the graft by mobilizing a segment of the transverse colon and delivering it through a window created in the transverse mesocolon into the infracolic compartment (Fig. 2e).

Postoperatively, the patient stayed in the ICU for 2 days. Blood culture and tissue culture from the aortic wall were free from bacteria. He was dismissed from the hospital on a postoperative day 10. Oral medications changed his antibiotics to nemonoxacin (0.5 g/day) for at least 6 months. CTA showed a patent abdominal aorta without pseudoaneurysm or any infection symptom after a 1-year follow-up (Fig. 2f).

## DISCUSSION

Infected infrarenal aortic pseudoaneurysm with AVF is an infrequent but severe complication and is unlikely to improve with conservative treatment. Open surgical repair is the most established option for the definitive eradication of the native aortic infection. Extraanatomical or *in situ* reconstruction with prosthetic grafts or homograft replacement is a common therapeutic alternative [2, 3]. Extraanatomical reconstruction has mostly been replaced by *in situ* reconstruction due to better patency. *In situ* reconstruction can achieve immediate limb revascularization, avoid groyne and axillary incisions and decrease long-term graft occlusion and amputation rates.

The most suitable graft material for repairing infected aorta remains controversial. Cryopreserved allografts or autologous deep femoral vein grafts are optimal alternatives with low risk of reinfection and better survival rates. However, the availability of allografts is the main limiting factor for usability [4, 5]. Harvesting autologous graft veins prolongs procedural time and may cause additional morbidity, and the size mismatch may cause aortic stenosis. The silver-coated or antibiotic-bonded grafts cannot be recommended based on the lack of biointegration. Under those circumstances, selecting a bovine pericardium patch is probably the better choice. This study confirms that the usability of bovine pericardium as a vascular prosthesis is a promising alternative. The main advantage is the shelf pericardial patches' simple, easy and quick availability, and all necessary grafts' sizes and lengths can be constructed. We used the two 8 cm × 10 cm pericardial patches in this study as a bifurcate graft, and one 3 cm × 4 cm patch is sufficient to treat the pseudoaneurysm and AVF. Moreover, no additional tissue incision is necessary compared with femoral vein grafting. The primary and critical elements of the procedure performed in this case were total infected tissues excision, debridement, *in situ* reconstructions of the aorta using pericardiac graft with great omental coverage and AVF repair to prevent leakage.

## CONCLUSION

*In situ* reconstruction with a self-made pericardial patch followed by prolonged antibiotic therapy was effective and safe for native aortic pseudoaneurysm with AVF.
